# Exploring the potential of mindfulness-based therapy in the prevention and treatment of neurodegenerative diseases based on molecular mechanism studies

**DOI:** 10.3389/fnins.2023.1097067

**Published:** 2023-06-13

**Authors:** Congcong Wu, Yue Feng

**Affiliations:** College of Acupuncture, Moxibustion, and Tuina, Chengdu University of Traditional Chinese Medicine, Chengdu, China

**Keywords:** mindfulness therapy, neurodegenerative disease, Parkinson’s disease, Alzheimer’s disease, amyotrophic lateral sclerosis, molecular mechanism

## Abstract

Neurodegenerative diseases (ND) have received increasing attention due to their irreversibility, but there is still no means to completely cure ND in clinical practice. Mindfulness therapy (MT), including Qigong, Tai Chi, meditation, and yoga, etc., has become an effective complementary treatment modality in solving clinical and subclinical problems due to its advantages of low side effects, less pain, and easy acceptance by patients. MT is primarily used to treat mental and emotional disorders. In recent years, evidence has shown that MT has a certain therapeutic effect on ND with a potential molecular basis. In this review, we summarize the pathogenesis and risk factors of Alzheimer’s disease (AD), Parkinson’s disease (PD), and amyotrophic lateral sclerosis (ALS), relating to telomerase activity, epigenetics, stress, and the pro-inflammatory transcription factor nuclear factor kappa B (NF-κB) mediated inflammatory response, and analyze the molecular mechanism basis of MT to prevent and treat ND, to provide possible explanations for the potential of MT treatments for ND.

## 1. Introduction

Neurodegenerative diseases (ND), primarily caused by the loss of specific neurons in the central nervous system, include AD, PD, ALS, and other disorders. The etiology is primarily linked to abnormal protein accumulation, gene mutation, increased reactive oxygen species, neuroinflammation, mitochondrial dysfunction, and apoptosis (Perry et al., 2010; Gómez-Gómez and Zapico, 2019; Hou et al., 2019; Irwin and Vitiello, 2019; Wu et al., 2019; Madore et al., 2020). ND primarily affects individuals over the age of 65, and symptoms generally worsen over time (Qiu and Fratiglioni, 2018). More than 10 million people worldwide now suffer from ND annually, and this number is rising each year along with the world’s aging population (Behl et al., 2021). While ND poses a great threat to human health, it also increases the burden on the healthcare system. There is currently no means to completely cure ND in clinical practice, and most drugs can only slow the rate of ND decline and improve the quality of patient survival. The possibility of traditional TCM non-drug therapy for ND is being studied in an increasing number of research studies as TCM gains popularity (Liu et al., 2022).

**Figure 1:**
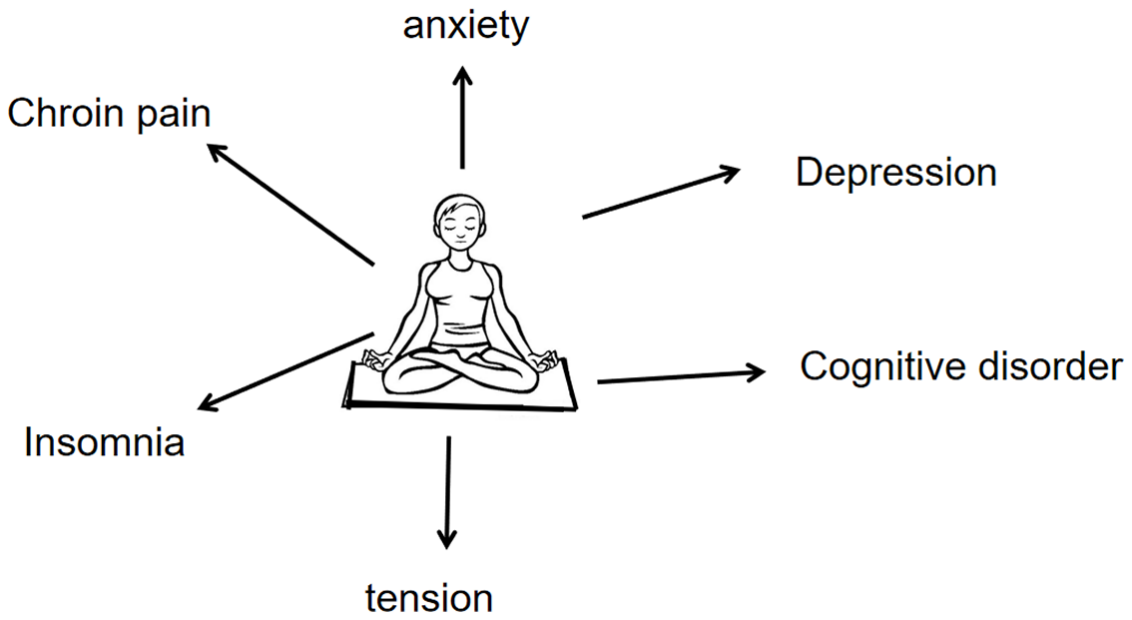
Diseases for which MT is currently used to treat.

MT includes qigong, Tai Chi, meditation, yoga, and other forms of physical and mental activities. Even 5,000 years ago, the ancient Chinese had mastered the self-exercise method for optimizing body and mind fitness through meditation and breath regulation. Tai Chi, meditation, yoga, and other forms of alternative medicine have progressively emerged. In a broad sense, mindfulness has been defined as a type of present-centered awareness that is unelaborate, nonjudgmental, and accepts every thought, feeling, or sensation as it arises in the attentional field (Bishop et al., 2004). The MT recommends that practitioners cultivate this awareness through meditation, actively and objectively attend to the present moment with an attitude of acceptance (not over-identification) rather than a reaction attitude, and concurrently collaborate with specific operations to achieve body and mind in order to achieve physical and mental balance. MT has been widely employed in the treatment of depression, anxiety, chronic pain, and sleep disturbances (Figure 1). Research conducted in 2013 by Gao and Xu discovered that long-term regular practice of Qigong exercise by the elderly may slow the rate of mental decline (Gao and Xu, 2013) and that MT has significant efficacy in the treatment of cognitive disorders (Rawtaer et al., 2015; Fam et al., 2020; Jin et al., 2020).

In recent years, the significance of MT in the prevention and treatment of ND has gotten a lot of attention (Dong et al., 2016; Song et al., 2017; Brasure et al., 2018; Kwok et al., 2019; Deuel and Seeberger, 2020; Guzman-Martinez et al., 2021), but its molecular mechanism has not been systematically summarized. Therefore, this paper begins with the proven molecular mechanism of MT and the risk factors for ND in order to investigate the potential molecular mechanism of MT to prevent ND.

## 2. Brief overview of ND

The term “ND” refers to a broad range of disabling and frequently unpredictable disease groupings that are all caused by neuronal loss and degeneration (Behl et al., 2021).

AD is the most common form of progressive ND, which is usually characterized by physical dysfunction, cognitive dysfunction, memory loss, and a progressive loss of self-care capacity. AD is the most common cause of dementia, accounting for 50–75% of all dementia patients (Yoshiyama et al., 2013). In the world, there are approximately 50 million people who have AD, and the frequency of the disease is increasing due to an aging population, according to a 2018 report from the Alzheimer’s Association. There will be 152 million people living with dementia (Livingston et al., 2020). Extracellular β-amyloid (Aβ) deposition and intracellular neurofibrillary tangles are the two pathologically distinctive abnormalities in AD (Yin et al., 2017). Among them, Aβ begins to build up nearly 20 years before dementia manifests, and the ensuing hard plaques interact with acetylcholine to cause inflammatory reactions and impair synaptic transmission, which may further cause the particular protein (tau) to degrade and exacerbate AD.

Neuroinflammation, neuronal loss and death, gliosis, synaptic loss, and impaired main synaptic function are pathogenic features of AD (Tönnies and Trushina, 2017). Along with atrophy in areas like the hippocampus, temporal lobe, parietal lobe, frontal cortex, and thalamus (Knight et al., 2016). AD is also linked to increased oxidative stress, which is considered to be a central factor in AD (Bai et al., 2022), dysregulated gene expression, cytokines, neurotrophins, and stress markers Telomere shortening and deterioration of brain connectivity are also linked to the disease’s pathophysiology (Ng et al., 2021).

After AD, PD is the most common ND and the most common severe mobility disability globally (de Lau and Breteler, 2006). It primarily affects older persons and affects 8–18 per 100,000 people annually (de Lau and Breteler, 2006). The prevalence of PD increases with age (Pringsheim et al., 2014). Dyskinesia, with progressive bradykinesia, rigidity, resting tremor, and abnormal posture and gait as its main manifestations, as well as non-motor disorders like hyposmia, constipation, sleep disturbance, depression, and cognitive impairment, are the main clinical characteristics of Parkinson’s disease (Greenamyre and Hastings, 2004). PD is a form of multisystem alpha-synucleinopathies characterized by selective loss of dopaminergic neurons and deposition of Lewy bodies in the substantia nigra, resulting in extensive involvement of other central nervous system structures and peripheral tissues (Cacabelos, 2017). Aging appears to be the only significant risk factor for PD development (Rokad et al., 2017). In addition, α-synuclein accumulation, mitochondrial dysfunction, autophagy impairment, and oxidative stress are common factors in PD pathogenesis. Factors such as oxidative stress play a central role in PD (de Lau and Breteler, 2006; Cacabelos, 2017).

ALS is a specific form of ND, but the etiology still remains unclear. ALS impacts the muscles and central nervous system (van Es et al., 2017). The majority of ALS patients, are middle-aged or elderly and above the age of 40. It is a deadly and somewhat uncommon ND. Approximately 3–6 persons out of every 100,000 people have ALS (Chiò et al., 2013). Motor neurons in the spinal cord, brain stem, and motor cortex are the main targets of ALS (Heiman-Patterson et al., 2015). Memory loss, cognitive decline, and decreased speech, swallowing, and respiratory function are all caused by neuronal death (Brown and Al-Chalabi, 2017). Statistics show that Cu/Zn superoxide dismutase gene mutations account for 20% of familial ALS cases (Heiman-Patterson et al., 2015). Aggregation and buildup of the ubiquitinated protein inclusion body TDP-43 in motor neurons are the neuropathological signs of ALS (van Es et al., 2017). The pathogenesis of ALS is also tightly linked to aging, oxidative stress (Aborode et al., 2022), RNA damage repair and axonal development, mitochondrial malfunction and autophagy, and acquired living conditions. And Oxidative stress is one major contributor to ALS pathogenesis. The result is an increased intracellular level of highly reactive free radicals, combined with defective antioxidant compensation systems that produce oxidative stress.

The aberrant buildup of cytoplasmic or nuclear proteins in the brain is the common pathogenic mechanism for various disorders, despite the fact that their etiologies and sites of lesions differ. Common risk factors include aging, genetic mutations, inflammation, and stress. Early treatment of these risk factors may lower the likelihood of developing ND and/or postpone the disease’s onset.

## 3. Molecular mechanism of MT

### 3.1. MT lengthens telomeres

MT appears to be a telomere-protective agent. Qigong (Baduanjin) (Tiwari et al., 2014), mindfulness meditation, and yoga (Schutte and Malouff, 2014; Rathore and Abraham, 2018; Dasanayaka et al., 2022) can lengthen telomeres and boost telomerase activity in both white blood cells and peripheral blood mononuclear cells (BPMC). Telomeres are eukaryotic chromosome ends that have repeated DNA sequences and unique cap-like structures that support chromosomal integrity. Telomeres are not fully reproduced because DNA polymerases are unable to complete the replication of the ends of linear molecules. As a result, telomeres shorten with each replication (Shay, 2018), and when they are sufficiently shortened, cells stall until senescence. Despite the fact that short telomeres are a pathogenic cause of senescence, telomerase re-expression can prevent premature senescence caused by telomerase deficiency and the specificity of short telomeres (Bär and Blasco, 2016).

Telomerase is the fundamental nuclear protein reverse transcriptase. Telomerase increases the amount of telomeric DNA at the ends of eukaryotic chromosomes to maintain the length of the telomeres (Epel et al., 2009). MT promotes telomeric DNA synthesis and cell division, counteracts telomere depletion due to cell division, and interrupts the telomere shortening process or lengthens telomeres by increasing telomerase activity and causing telomerase re-expression (Smith et al., 2020). Telomerase re-expression lengthens telomeres, guards against fusion and degeneration of chromosome ends, and is crucial for chromosome placement, replication, and protection, as well as for the regulation of cell growth and lifespan (Anitha et al., 2019).

### 3.2. MT and DNA methylation

One of the most well researched epigenetic processes, DNA methylation modifies chromatin structure without changing nucleotide base sequences. Particularly in the human brain and blood (García-Campayo et al., 2018), DNA methylation is crucial for controlling the expression of genes (Martínez-González et al., 2020). Long-term MT can methylate genes including FKBP5 (Bishop et al., 2018), SCL6A4 (Stoffel et al., 2019), NR4A2 (García-Campayo et al., 2018), and CLU (Huang et al., 2016), influencing the proteins encoded by these genes, controlling the dynamic process of methylation and demethylation, and enhancing the organism’s benefit from the epigenetic process.

### 3.3. MT and inflammation

Numerous trials have demonstrated that yoga, Tai Chi, and meditation practices can prevent the accumulation of ROS in cells by upregulating the activity of ROS-degrading enzymes through meditation with rhythmic breathing exercises and reducing oxidative stress markers such as ROS and 8-hydroxy-2-deoxyguanosine (Huang et al., 2014; Kumar et al., 2015; Gagrani et al., 2018) and maintaining brain homeostasis. Furthermore, meditation (Wetherell et al., 2017), Tai Chi and qigong (Campo et al., 2015; Klein et al., 2016; Larkey et al., 2016) can reduce stress and lower salivary cortisol levels in people with high baseline levels (Moraes et al., 2018), while having no effect on baseline levels in the normal population. A randomized clinical trial also confirmed that long-term mindfulness training can reduce the accumulation of cortisol and saliva in the hair (Puhlmann et al., 2021), resulting in increased immune responsiveness (Tang et al., 2009; Svetlov et al., 2019). In addition, short-term meditation has been shown to increase side-sympathetic nerve tension and decrease the activity of the sympathetic nervous system. While the reaction of the sympathetic nervous system can drive inflammation, the side neurosympathetic system can inhibit NF-κB and inflammatory reactions by activating acetylcholine (Haroon et al., 2012).

Several studies using gene expression analysis MT have identified downregulation of NF-κB target genes, which can be interpreted as a reversal of the molecular signature of chronic stress effects (Buric et al., 2017). Several randomized controlled trials have confirmed that the expression of the pro-inflammatory genes RIPK2 and COX2 (Kaliman et al., 2014), as well as NF-κB activity, are significantly reduced in PBMC in long-term meditators (Creswell et al., 2012; Black et al., 2015; Bower et al., 2015). In addition, Tai Chi (Irwin et al., 2014; Buric et al., 2017), and yoga (Bower et al., 2015) also reduced levels of the inflammatory markers C-reactive protein (CRP), tumor necrosis factor (TNF-α), and interleukin 6 (IL-6), and had downregulation of several genes involved in leukocyte production and inflammation (Buric et al., 2017).

## 4. Possible therapeutic mechanisms of MT against ND

### 4.1. MT delays aging and has the potential to combat ND cognitive impairment

The largest risk factor for the majority of ND is aging. Each lower motor unit cell type is prone to its own unique collection of aging-related phenotypes that may exacerbate the course of the ND disease (Guo and Yu, 2019; Hou et al., 2019; Pandya and Patani, 2020; Liu, 2022). The physiological process of aging produces a multitude of molecular and cellular abnormalities. DNA damage, mitochondrial dysfunction, telomere length loss, and oxidative stress are the four main causes of aging (Wyss-Coray, 2016; Hernandez-Segura et al., 2018), and telomere loss is thought to be the primary cause of aging. Age-related cognitive decline is also linked to shorter telomeres (Bär and Blasco, 2016), and Mendelian randomization research also found a causal link between shorter telomeres and increased risk of AD (Guo and Yu, 2019).

MT delays cellular senescence by increasing telomerase activity to extend telomeres (Campisi and d'Adda di Fagagna, 2007; Blackburn et al., 2015). Genetically or pharmacologically, the reduction of senescent cells improves Aβ peptide and tau protein-induced neuropathology and improves memory in AD model mice (Bussian et al., 2018; Zhang et al., 2019), thus achieving a counteracting effect on cognitive impairment (Hou et al., 2019). Notably, studies have confirmed increased gray matter volume and significantly reduced brain atrophy in the hippocampus and prefrontal cortex in expert meditators (aged 22–77) (Chételat et al., 2017), and Wolkowitz et al. hypothesized that BPMC telomerase activity may correlate with hippocampal enzyme activity and hippocampal volume (Wolkowitz et al., 2015; Deng et al., 2016). Then we can venture to speculate that meditation may have the effect of treating cognitive impairment and promoting memory by increasing telomerase activity and increasing prefrontal cortex gray matter volume as well as hippocampal volume (Cheng et al., 2013), thus reducing the risk of developing AD and/or delaying disease onset. However, this hypothesis is currently controversial and needs further validation.

### 4.2. The epigenetic potential of ND is controlled by MT

Epigenetic dysregulation can lead to cognitive impairment and neuronal death associated with ND (Hwang et al., 2017).

Long-term meditators’ genome-wide alterations in DNA methylation were examined by García-Campayo et al. (2018). The 64 differently methylated areas that meditators produced compared to non-meditators belong to 43 genes, and 48.4% of these regions were determined to be directly related to common human disorders, of which 9 (14%) were in genes linked to ND (AD, PD, and ALS) (García-Campayo et al., 2018). And among these related genes, nuclear receptor family 4 group A member 2 (Nr4a2) is the most differentially methylated. This gene encodes a nuclear transcriptional regulator that has been identified as a key regulator of dopaminergic (DA) neuronal differentiation, survival, and maintenance and as being essential for neuronal development, particularly for the maintenance of the DA system (Jakaria et al., 2019).

Nr4a2 prevents inflammation-mediated DA neuron death and is crucial for hippocampus synaptic plasticity and memory formation (Català-Solsona et al., 2021). It may be possible to treat DA dysfunction-related disorders like PD by promoting the methylation of this gene through meditation. It’s interesting to note that new research has discovered that altered Nr4a2 expression is likewise linked to the course of AD, and Nr4a2 agonists can speed up the degradation of Aβ by considerably reducing γ-secretase activity by upregulating an Aβ-degrading enzyme (insulin-degrading enzyme). The characteristic AD symptoms were significantly reduced in the agonist-treated mouse model of AD, and cognitive performance was significantly enhanced (Jakaria et al., 2019; Moon et al., 2019).

Additionally, the CpG sites of FKBP5 GREs in intron 7 and the promoter region speed up age-related demethylation in AD patients, increasing the expression of FKBP51 mRNA and protein with aging. Tau cannot be separated into less toxic tangles due to FKBP51’s interference with tau degradation and promotion of tau oligomer formation. This increases neurotoxic tau, which advances AD (Blair et al., 2013). Long-term meditation increases FKBP5 DNA methylation and decreases FKBP51 expression, which reduces tau neurotoxicity and slows the course of AD. In contrast, decreased FKBP5 DNA methylation increases FKBP51 expression.

Epel et al. also discovered that CLU gene expression and PSEN1 gene expression were both decreased following meditation (Epel et al., 2016; Huang et al., 2016). Reduced CLU gene expression can lower the risk of AD and PD, according to genome-wide correlation studies that have identified the CLU gene as a well-established risk gene related to AD and PD (Karch and Goate, 2015; Lin et al., 2021). Although PSEN1 encodes the -secretase necessary for the synthesis of Aβ peptides, PSEN1’s decreased expression leads to insufficient synthesis of the γ-secretase complex, which may be caused by a decrease in the synthesis of Aβ peptides as a result of meditation and a relative decrease in the γ-secretase needed for the hydrolysis of Aβ peptides.

By examining blood markers after meditation, Epel et al. also discovered that the level of Aβ_40_ in the blood of meditators decreased (Epel et al., 2016). Since CLU induces the deposition and removal of Aβ (Maturana-Candelas et al., 2021), meditation may lower the level of Aβ_40_ by reducing the expression of the CLU gene, thereby lowering the Aβ_42_ /Aβ_40_ ratio and lowering the risk of AD (Chouraki et al., 2015). The epigenetic modifications in subtelomeric areas may be related to telomere length, and DNA methylation may also be implicated in the stability of telomere length in long-term meditators’ specific subtelomeric regions (Mendioroz et al., 2020).

Long-term meditators, compared to non-meditators, have different areas that are methylated in pathways related to cellular senescence, neurotransmission, lipid and glucose metabolism, immunology, and inflammation (Kaliman, 2019). These genes’ methylation may affect ND directly or indirectly. Reduced Aβ deposition has a protective or mitigating effect on cognitive impairment caused by AD and PD. This effect is due to the methylation of ND-related genes resulting from MT, such as the Nr4a2 gene and the CLU gene, whose expression is strongly associated with the deposition and clearance of Aβ and tau.

Through the epigenetic process of DNA methylation, meditation may control the expression of ND-related genes, alleviating symptoms and slowing the course of the disease. However, long-term meditation accumulation might be necessary to regulate gene expression, which short-term meditation cannot do. This hypothesis has to be proven in trials using larger sample sizes.

### 4.3. MT’s potential to combat ND-related neuroinflammation

Chronic oxidative stress results in the accumulation of reactive oxygen species, which damages target molecules like DNA, proteins, and lipid structures. An imbalance in the antioxidant system is one of the key mechanisms causing ND (Tönnies and Trushina, 2017). The antioxidant system may become unbalanced, the brain’s equilibrium may be lost, and ND may result if the balance between the production and consumption of reactive oxygen species is upset (Radi et al., 2014; Nissanka and Moraes, 2018; Stefanatos and Sanz, 2018; Collin, 2019; Yeung et al., 2021).

Chronic stress may promote the loss of nigrostriatal cells in PD, hastening the disease’s course (van der Heide et al., 2021), and stressful conditions may intensify the condition’s motor symptoms, such as tremor.

Additionally, in human ALS fibroblasts and pluripotent stem cell iPSC-motoneurons, prolonged stress stimulates the production of stress granules and pathogenic TDP-43 aggregates, accelerating the course of ALS (Ratti et al., 2020).

Stress also leads to a reduction in hippocampal volume and a decrease in the number of glucocorticoid receptors (GR) in the hippocampus (Frodl and O’Keane, 2013), as well as stimulation of the hypothalamic–pituitary–adrenal (HPA) axis and sympathetic nervous system, which promotes the release of glucocorticoids (GC) and catecholamines. The decrease in the number of GR and increased GC release leads to elevated GC levels and brain atrophy, such as in the hippocampus, due to prolonged high levels of GC stimulation, which puts the person in a state of extreme stress and anxiety and exacerbates the progression of AD (Boutrup et al., 2019).

In addition, high levels of GC initiate an immune response in brain microglia, making them pro-inflammatory and promoting a neurotoxic response (Milligan Armstrong et al., 2021). In an AD rat experiment, it was demonstrated that Aβ_25-35_ amyloid toxicity affects the adaptive response of the HPA axis to stress (Brureau et al., 2013), resulting in chronically high levels of cortisol in patients (Vyas and Maatouk, 2013).

The HPA axis is a crucial neuroendocrine signaling system that regulates physiological homeostasis and stress reactions. It is a well-known characteristic of AD to have a very active HPA axis (Notarianni, 2013), which is indicated by excessive cortisol output. The HPA axis’s overproduction of cortisol affects somatic tissues through blood flow, and elevated levels of GC, including cortisol, lead to hemodynamic, endocrine, and immune system problems as well as increased accumulation of Aβ and tau, which cause increased brain atrophy, behavioral deficits, mood disorders (Du and Pang, 2015), and/or cognitive decline (Milligan Armstrong et al., 2021), all of which accelerate the progression of AD and PD (de Pablos et al., 2014; Ennis et al., 2017).

Additionally, it has been demonstrated that chronic inflammation raises the risk of ND (Buric et al., 2017). It is well established that the nuclear factor NF-κB, a transcription factor that promotes inflammation, can be inhibited by the cortisol-GR complex, preventing the transcription of genes that promote inflammation. NF-κB can transcriptionally regulate the expression of cytokines. The transcription factor is closely linked to mammalian aging, inflammation, and stress. As a molecular indicator of chronic stress (Buric et al., 2017), the upregulation of pro-inflammatory genes is thought to cause dysregulation of GC secretion, decreased sensitivity to GR in the brain and immune cells, and a lack of suppression of NF-κB-mediated inflammation (Boutrup et al., 2019). This vicious cycle ultimately increases the risk of developing ND.

Neuroinflammation is associated with ND and is one of the important mechanisms in the development of ND (Kwon and Koh, 2020), and both central inflammation and systemic inflammation can accelerate the progression of ND (Perry et al., 2010; Shih et al., 2015). MT delays aging due to DNA damage by inhibiting NF-κB activity (Tilstra et al., 2012), which in turn delays aging-related neurodegeneration (Tilstra et al., 2012; Álvarez-López et al., 2022).

**Figure 2:**
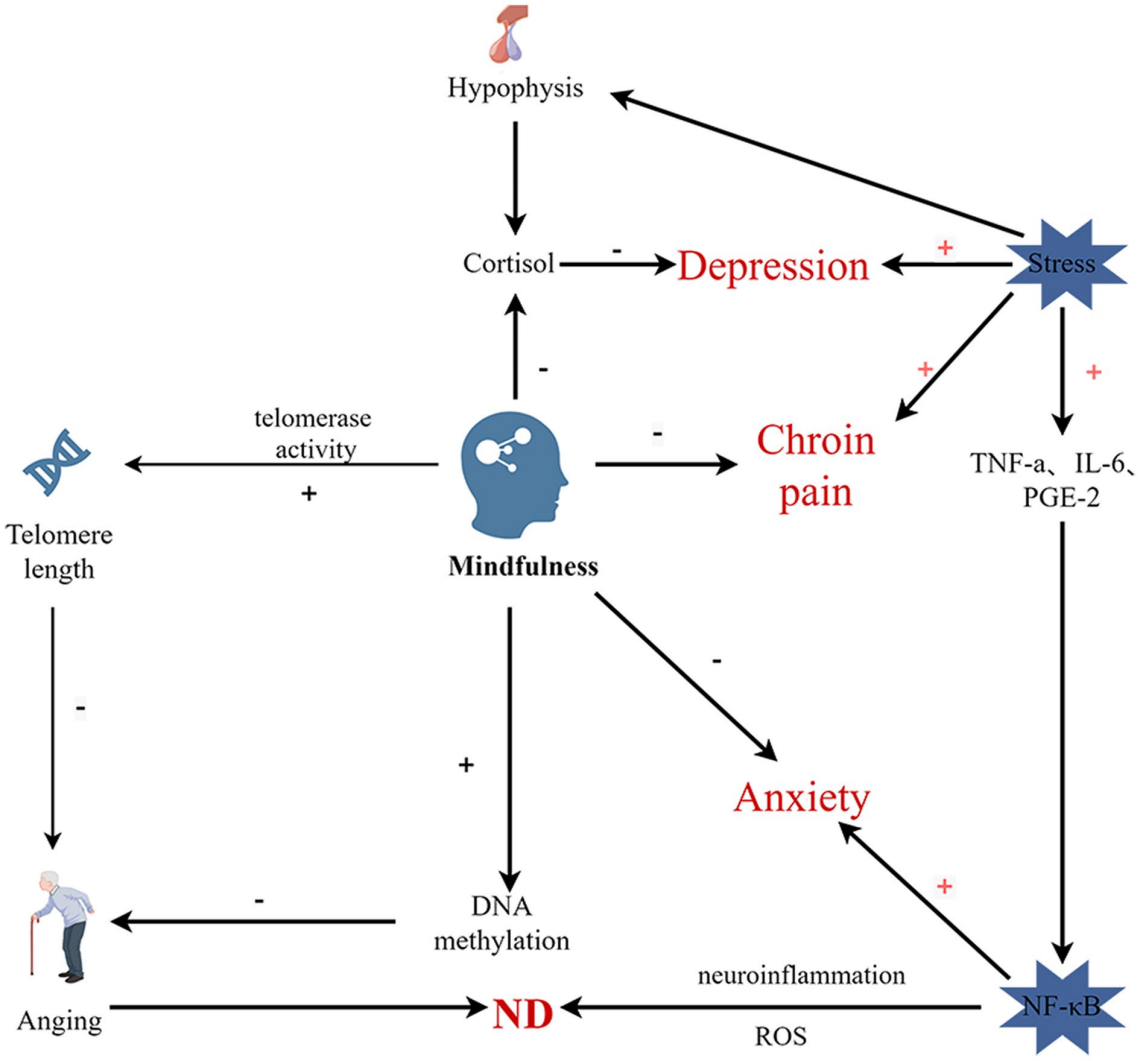
Schematic diagram of hypothesized pathway of the effects of MT on mental and physical wellbeing and aging. NF-kB-Nuclear Factor kappa B; IL-6-Interleukin-6; TNF-α-Tumor Necrosis Factor-alpha; PGE-2-prostaglandin E2; ROS-Reactive Oxygen Species. Stress activates the HPV axis, releasing cortisol, and increased cortisol and oxidative stress lead to increased stress. MT can prevent and treat ND by promoting telomerase activity, thereby lengthening telomeres and slowing aging, and by promoting DNA methylation associated with ND and reducing neuroinflammation (By Figdraw).

Long-term practice of meditation, yoga, Tai Chi, and qigong induces downregulation of NF-κB-related upstream and downstream targets, which may reduce stress and inflammatory responses by inhibiting the NF-κB pathway through the RIPK2 pathway (Bhasin et al., 2013), delaying the onset of ND. Animal studies have shown that acute activation of pro-inflammatory cytokine signaling in the brain in response to peripheral immune activation is associated with deficits in hippocampal-dependent memory (Dantzer et al., 2008). MT reduction in stress can reduce the hyperactivity of the HPA axis, resulting in a decrease in the level of GC accumulated in the nuclei of neurons in different brain regions, especially the hippocampus, hypothalamus, and amygdala, which are rich in GR. Brain shrinkage and memory impairment may be delayed as a result of MT’s lowering of pro-inflammatory cytokines. Regular MT training can have beneficial effects on ND by improving neuroendocrine stress responses, improving HPA axis stress responses (Khalsa, 2015), and nuclear receptor-mediated transcriptional changes to reduce neuroinflammation (Figure 2).

## 5. Discussion

Current research finds that the mechanisms affecting the onset and development of diseases mainly include: incorrect folding and aggregation of proteins, neuropathy, cellular procedural death and aging. Circadian rhythm disorder, nutritional inadequacies, stress, inflammatory reactions, age, and gene mutations are typical risk factors for ND (Perry et al., 2010; Gómez-Gómez and Zapico, 2019; Hou et al., 2019; Irwin and Vitiello, 2019; Wu et al., 2019; Madore et al., 2020). MT can intervene in these risk variables to prevent and treat ND. By increasing telomerase activity to postpone aging, lowering anxiety and depression to regulate circadian rhythms (Yingwei et al., 2019), lowering stress and NF-κB-induced neuroinflammatory responses, and changing DNA methylation relevant to ND to regulate gene expression, MT can prevent and reduce the progression of ND. In turn, these processes engage in mutually beneficial interactions. Telomerase, for instance, possesses antioxidant, anti-apoptotic, neurotrophic, and neurogenesis-promoting properties that help restore brain cell suppleness and viability in addition to lengthening telomeres to delay aging. Additionally, oxidative stress and inflammation can shorten telomeres and speed up aging (Bär and Blasco, 2016). Antioxidants have neuroprotective effects (Hou et al., 2019), and MT can reduce the occurrence of neuroinflammation and slow down the aging process by reducing oxidative stress. Meanwhile, bioinformatic analysis predicted that epigenetic responses to MT exercises may regulate inflammatory pathways dependent on tumor necrosis factor α and NF-κB signaling. Through these mechanisms, MT can create a positive cycle that will improve the symptoms of ND and slow the ND process.

The clinical effects of MT on ND have demonstrated that MT can enhance patients’ quality of life, improve symptoms like anxiety and depression, and slow the progression of the disease. However, the majority of these trials evaluated the effectiveness using a scale. According to the current study of mind-based brain area research, inflammatory factor research, and the subjective score table (Newberg et al., 2014; Lou, 2017; Kwok et al., 2019), and less frequently with biological markers. Extensive experimental research is still needed to understand the mechanism of MT activity in ND. Future research should explore if MT intervention at a younger age would lower the incidence of ND. More clinical trials are also required to determine whether traditional Chinese medicine offers unique, superior benefits for ND prevention and treatment. Mind-based physical and mental therapy is an interesting and beneficial way, in the modern society, where public health awareness is generally enhanced, early and continuous mind intervention will play a role in disease with age to prevent and delay illness, and it is worth our study.

## Author contributions

CW drafted the manuscript. YF revised the manuscript. All authors contributed to the article and approved the submitted version.

## Funding

This research was supported by the National Natural Science Foundation of China (grant number: 82074575).

## Conflict of interest

The authors declare that the research was conducted in the absence of any commercial or financial relationships that could be construed as a potential conflict of interest.

## Publisher’s note

All claims expressed in this article are solely those of the authors and do not necessarily represent those of their affiliated organizations, or those of the publisher, the editors and the reviewers. Any product that may be evaluated in this article, or claim that may be made by its manufacturer, is not guaranteed or endorsed by the publisher.
